# Assessment of stigma in patients with cystic fibrosis

**DOI:** 10.1186/1471-2466-14-76

**Published:** 2014-05-01

**Authors:** Smita Pakhale, Michael Armstrong, Crystal Holly, Rojiemiahd Edjoc, Ena Gaudet, Shawn Aaron, Giorgio Tasca, William Cameron, Louise Balfour

**Affiliations:** 1 Ottawa Hospital Research Institute, 501 Smyth Road, K1H 8 M5 Ottawa, Ontario, Canada; 2 The Ottawa Hospital, 501 Smyth Road, K1H 8 M5 Ottawa, Ontario, Canada; 3 The University of Ottawa, 451 Smyth Road, K1H 8 M5 Ottawa, Ontario, Canada; 4 Division of Respiratory Medicine, The Ottawa Hospital, 501 Smyth Road, Ottawa, Ontario K1H 8 L6, Canada

**Keywords:** Psychometric, Treatment Adherence, Validation, Focus group

## Abstract

**Background:**

Research that explores stigma in Cystic Fibrosis (CF) is limited. Productive cough, repeated lung infections, and periods of serious illness requiring hospitalizations are among common symptoms of CF. These symptoms may cause a negative perception by others. We developed a CF-specific Stigma Scale and tested its psychometric properties.

**Methods:**

We conducted a focus group with 11 participants including adult patients with CF (*n* = 5) and their informal caregivers (*n* = 6). The thematic content of the focus group was analyzed to find key themes. We developed a CF-specific Stigma Scale and assessed its psychometric properties in a 3-month prospective cohort study of adult CF outpatients (*n* = 45).

**Results:**

Stigma emerged as consistent concern for people living and caring for those with CF, affecting both patients’ lives and health through the focus group. Using the newly developed CF Stigma scale, the mean baseline score was 16.6 (SD = 4.5, Range = 10-25). The CF Stigma Scale demonstrated robust psychometric properties: 1) Internal consistency: α = 0.79; 2) Mean inter-item correlation: 0.30 with good test-retest reliability; 3) Convergent validity: Positive associations with depression, severity of CF symptoms and anxiety; negative associations with validated quality of life scores were observed.

**Conclusions:**

Stigma is measurable and significantly impacts the lives of CF patients. Further research should investigate the role of stigma in patients living with CF.

## Background

Cystic Fibrosis (CF) is an inherited, chronic, progressive and fatal disease [1]. Common symptoms—including productive cough, repeated lung infections, and periods of serious illness requiring hospitalizations—may cause a negative perception by others [2-4]. For patients with CF, the physical impact, premature mortality, and social unacceptability of symptoms may be stigmatizing [5]. People living with CF report difficulties with psychological adjustment and quality of life [6,7] and may experience emotional disturbances, low self-esteem, feelings of helplessness, and depression [8]. Importantly, psychosocial functioning has been linked to impaired pulmonary function in CF patients [9].

Health-related stigma is defined as a personal experience with specific characteristics including: exclusion, rejection, blame, or devaluation resulting from the anticipation of a negative judgment [10]. Stigma has been demonstrated to have a significant impact on overall health outcomes and quality of life [11]. This stigma can interfere with a patient’s adherence to the intensive treatment regimens required for chronic conditions such as Human Immunodeficiency Virus/Acquired Immune Deficiency Syndrome (HIV/AIDS), Hepatitis C, diabetes, and lung cancer [10,12,13]. For individuals living with CF, stigma could be associated with lower adherence to their treatment regimen.

There is very limited research exploring stigma associated with CF. Pizzignacco and colleagues hypothesize that in CF patients, stigma could be associated with decreased social contact, impaired relationships, and worsening of disease related to decreased morale and this could lead to a deterioration in lung function, weight and physical function [14]. The current study was designed to investigate the relationship between stigma and CF. The objectives of this study were: 1) to explore if CF patients experience stigma, 2) to develop/adapt a scale to measure stigma in CF, and 3) to assess the adapted stigma scale’s psychometric properties in a prospective, cross-sectional cohort of adult CF patients. We hypothesized that CF patients will experience significant health-related stigma measurable by a CF stigma scale.

## Methods

This study is a part of a larger project entitled, “The Ottawa Cystic Fibrosis Treatment Knowledge and Adherence Program”. We employed a mixed-method design with two phases: A qualitative phase to better understand the experience of individuals living with CF and a quantitative phase to measure stigma in individuals living with CF and to test the psychometric properties of the scale.

All study procedures (qualitative and quantitative phase) were approved by the Research Ethics Board of the Ottawa Hospital Research Institute, Ottawa, Canada. Written informed consent was obtained from all study participants.

### Qualitative phase

#### Focus group

A semi-structured, two-hour focus group was conducted with 11 adults (5 individuals living with CF and their caregivers) as part of a larger program of research investigating people caring for and living with CF. The focus group size was selected such that it was large enough to gain a variety of perspectives and small enough not to become disorderly or fragmented. This focus group obtained accounts of their experience with daily challenges, stigma, and managing and maintaining adherence to their complex treatment regimen. We used open-ended questions related to broad categories based on our clinical knowledge and experience to generate data based on the synergy of the group interaction. Importantly, the participants did not know each other, thus encouraging more honest and spontaneous expression of views and a wider range of responses. Data were audiotape recorded, transcribed verbatim, and analyzed using Thematic Analysis as outlined by Braun & Clarke, 2006 [15]. Thematic Analysis included identifying, analyzing and reporting patterns (themes) within data in order to minimally organize and describe our dataset in greater detail. We ensured that the themes were coherent, consistent and distinctive. Themes were then analyzed not just describing but balancing between analytic narrative and extracts.

#### Sample size and demographics of focus group participants

Of the 5 participants with CF, 2 were female; all were Caucasian with post-secondary education and aged 23 to 56 years. Three were employed full time, one part-time, and the other was on disability. Five of the six caregivers provided demographic data. They were all female, aged 24 to 55 years, with post-secondary education. We strictly followed the infection control procedures suggested by the Canadian Cystic Fibrosis Foundation during the focus group meeting [16].

#### Results

Preliminary themes identified from the focus group included: 1) integrating demands from social, occupational, and family responsibilities, 2) factors that improve or impede adherence (e.g. lifestyle, social support, personality style, work), 3) disclosure of CF, 4) navigating the health care system (e.g. drug insurance, transitioning through health care providers, fertility treatments), and 5) lack of knowledge about CF in the general public (e.g. stigma). Stigma emerged as a consistent concern during the focus group discussion. Most CF patients in the group noted that CF symptoms (e.g. coughing) negatively affected the general public’s reactions. Misconceptions about CF (e.g. increased lifespan expectations of CF patients in recent years), lack of awareness (e.g. school’s negative view of high calorie lunches) and extreme reactions (e.g. sent home from school for CF cough) were common.

The qualitative phase of the study highlighted the importance of stigma in individuals living with CF. A literature review revealed no scales to measure CF-specific stigma. As such, we adapted questions (with permission) from a well-validated scale, originally designed for HIV patients [17]. Participants also completed a CF symptom scale (Pakhale et al., unpublished work).

#### The adapted CF stigma scale

We preserved the original format of the HIV brief stigma scale [18] owing to its well tested psychometric properties. The wide ranges of stigma beliefs measured by the HIV stigma scale are derived from stigmatization theory. Item responses (“strongly disagree” to “strongly agree”) are recorded on a 4-point Likert scale, with higher agreement reflecting endorsement of stigma. Theoretical scores range from 10 to 40. High internal consistency for the subscales, ranging between 0.72 and 0.84, is established [18]. All the HIV items were retained for the CF scale, with the term ‘CF’ substituted for ‘HIV’ (e.g. “I have been hurt by how people reacted to learning I have HIV” to “I have been hurt by how people reacted to learning I have CF”). Each question in the 10-item scale falls into 1 of 4 domains: Personalized stigma, Disclosure, Public attitudes and Negative self-image.

### Quantitative phase

#### Methods

We conducted an observational cohort study at a multi-disciplinary adult CF outpatient clinic in Ottawa, Canada. Participants living with CF were eligible if they were 18 years of age or older, expected to continue receiving care at the CF clinic for the duration of the study (1 year), were English speaking and were willing and able to complete study questionnaires. Participants were excluded if they had an expected survival of < 1 year as per CF treatment guidelines or were lung transplant recipients. A psychological questionnaire package was administered to participants during a regular clinic visit and a follow-up was conducted at 3 months.

#### Sample size and demographics of prospective cross-sectional study

We approached 52 eligible participants; of these, 87% (*n* = 45) agreed to participate in the study. Baseline characteristics of the 45 study participants are presented in Table 1.

**Table 1:** Study cohort characteristics

**Variable**	**Total CF cohort N = 45 (%)**
Age (mean ± SD)	30.73 ± 10.80
Sex	
Female	19 (42.2)
Ethnicity	
Caucasian	42 (93.3)
Other	3 (6.7)
Education	
Secondary	9 (20.0)
Some university/college	10 (22.2)
University or more	26 (57.8)
Employment status	
Full-time	22 (48.9)
Part-time	5 (11.1)
Disability	7 (15.6)
Unemployed/Retired/Other	11 (24.4)
Drug insurance coverage	
Public	15 (33.3)
Private	22 (55.0)
None or missing	8 (11.7)
Living status	
Alone	6 (13.3)
Parents/family	15 (33.3)
Spouse/partner	20 (44.4)
Roommate	4 (8.9)
Relationship status	
Single	17 (37.8)
Married/Steady partner	27 (60)
Divorced	1 (2.2)

#### Recruitment and study questionnaires

The psychological questionnaire package included the following validated measures:

*The Short Form (SF) -12v2*[19]. The SF12v2 is a well validated self-report measure of quality of life (QoL). It consists of 12-items that ask patients to rate their current mental and physical health functioning in the past 4 weeks on 5-point scales (e.g. “all of the time” to “none of the time” and “excellent” to “poor”). Higher scores indicate greater level of QoL. The SF-12v2 has been found to have good internal consistency with reliability coefficients in the ranges of 0.82 to 0.87 for the PCS and 0.70 to 0.84 for the MCS in samples of people living with chronic illnesses (e.g. diabetes, congestive heart failure) [19].

*The CF-specific Quality of Life Questionnaire (CF-specific QoL)*[20]. The CF QoL is a 52-item self-report measure that assesses both physical and mental health as well as activities of daily living affected by CF over the past 2 weeks on a 6-point scale (“never” to “all of the time”).

*The Centre for Epidemiologic Studies Depression (CES-D)*[21]. The CES-D is a well-validated and widely used 20-item self-report measure that asks participants to rate the frequency with which they have experienced symptoms of depression over the past week ranging from “never” to “almost every day” (e.g. “I felt that everything I did was an effort”).

*The Generalized Anxiety Disorder Questionnaire (GAD) -7*[22]. The GAD is a 7-item validated measure used to assess anxiety in the past two weeks.

### Statistical analysis

Analyses were conducted using the Statistical Package for Social Sciences (SPSS) software (v18). Data were screened and statistical assumptions evaluated [23]. We did not impute missing data; complete case analysis was performed. Statistical significance was set at *p* = 0.05 (two-tailed) for all analyses. Mean and standard deviation (SD) of CF stigma scores were assessed at baseline and at 3 months. Psychometric properties of the CF Stigma Scale were analyzed using the baseline and 3 month data. We evaluated face and content validity, test-retest reliability and convergent validity. Two types of reliability were estimated on the total score and subscales: Internal consistency using Cronbach’s alpha coefficients and test-retest reliability [24]. The latter was calculated by comparing the consistency of scoring of the new CF Stigma Scale administered on two occasions (baseline and at 3 months) using a paired samples t-test and Pearson correlations. Based on the observed relationships between CF symptoms and quality of life, we conducted post-hoc tests that consisted of a mediational model hypothesizing that experiencing more symptoms leads to a reduced quality of life *because of* the simultaneous experience of stigma associated with CF symptoms. Due to the relatively small sample size of this study, we used bootstrapping (1000 resamples) to reliably estimate indirect effects, and avoid concerns regarding requisite assumptions for common parametric tests such as regression. The Preacher and Hayes SPSS Macro for Simple Mediation was used for this analysis [25]. A student t-test was performed to compare the stigma scale scores in our adapted CF Stigma Scale to the HIV Stigma Scale scores published by Logie and colleagues [26].

## Results

The CF Stigma Scale scores are depicted in Table 2. Mean CF stigma scores were 16.6 (SD = 4.5, Range = 10–25) at baseline and 16.9 (SD = 5.1, Range = 10–28) at follow-up. The CF Stigma Scale exhibited promising psychometric properties including very good test-retest reliability over a 3 month period and excellent construct validity (Table 3). No items demonstrated ceiling effects, and a satisfactory range and distribution of item responses was observed. In terms of reliability (Table 3), the CF Stigma Scale demonstrated adequate internal consistency, with a coefficient alpha of 0.79, and a mean inter-item correlation of 0.30 (*n* = 44). Three month test-retest correlations (*n* = 35) were good (*r* = 0.780, *p* < 0.001). A paired sample t-test revealed there were no significant differences between responses to items at study baseline (M = 16.6 ± 4.5) and at time two (M = 16.9 ± 5.1), *t* (33) = -1.065, *p* = 0.30). There was evidence of strong convergent validity (Table 3). The CF Stigma Scale scores correlated positively with symptoms of depression (*n* = 44, *r* = 0.529, *p* < 0.001), generalized anxiety (*n* = 39, *r* = 0.371, *p* = 0.014), and the severity of CF symptoms (*n* = 44, *r* = 0.479, *p* = 0.001). The CF Stigma Scale score was negatively correlated with generic quality of life scores (*n* = 44, *r* = -0.454, *p* = 0.002) and CF-specific quality of life scores (*n* = 39, *r* = -0.645, *p* < 0.001).

**Table 2:** The baseline CF stigma scale scores

	**Strongly disagree = 1**	**Disagree = 2**	**Agree = 3**	**Strongly agree = 4**
**N = 45**	**N (%)**	**N (%)**	**N (%)**	**N (%)**
1. I am very careful who I tell that I have CF	6 (13.3)	17 (37.8)	10 (22.2)	11 (24.4)
3. Having CF makes me feel unclean	30 (66.7)	7 (15.6)	6 (13.3)	1 (2.2)
4. Having CF makes me feel that I’m a bad person	37 (82.2)	6 (13.3)	1 (2.2)	-
5. Most people think that a person with CF is disgusting	25 (55.6)	13 (28.9)	6 (13.3)	-
6. Most people with CF are rejected when others find out	23 (51.1)	19 (42.2)	2 (4.4)	-
7. I have been hurt by how people reacted to learning I have CF	22 (48.9)	11 (24.4)	2 (4.4)	1 (2.2)
8. I have stopped socializing with some people because of their reactions to my having CF	23 (51.1)	13 (28.9)	7 (15.6)	-
10. I worry that people who know I have CF will tell others	23 (51.1)	15 (33.3)	4 (8.9)	2 (4.4)

Note: Higher agreement reflects higher stigma experienced by patient.

**Table 3:** Psychometric properties of the newly adapted stigma scale

**Validity**	**Correlations**		
CES-D (*n, r*, p-value)	*n* = 44, *r* = 0.529, *p* < .001		
GAD-7 (*n, r,* p-value)	*n* = 39, *r* = 0.371, *p* = .014		
CF symptom (*n, r,* p-value)	*n* = 44, *r* = 0.479, *p* = .001		
SF- 12 (*n, r*, p-value)	*n* = 44, *r* = -.454, *p* = .002		
CF-specific QOL (*n, r*, p-value)	*n* = 39, *r* = -.645, *p* < .001		
Reliability			
Internal consistency (α)	0.79		
Mean Inter-item correlation (mean, N)	0.30 (44)		
Three month test-retest (*r,* p-value)	*r =* 0.78; p < .001		
	Baseline	Follow-up	p-value
Mean differences between response items (mean, SD)	16.6 (4.5)	16.9 (5.1)	0.3

Center for Epidemiologic Study-Depression scale (CES-D).

Cystic Fibrosis symptom scale (CF symptom).

General Anxiety Disorder -7 scale (GAD-7).

Short Form (SF)-12.

Cystic Fibrosis-specific Quality of Life (CF-specific QOL).

### Post hoc tests: stigma as a mediator of symptoms and quality of life

**Figure 1:**
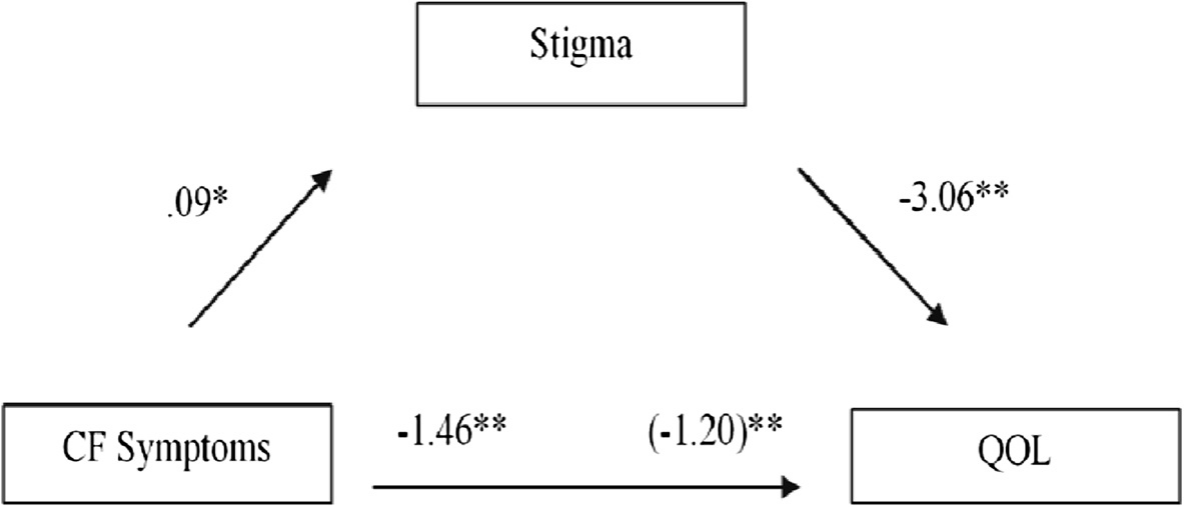
**Unstandardized regression coefficients for the relationship between CF symptoms and quality of life as mediated by stigma.** The unstandardized regression coefficient between CF symptoms and quality of life, controlling for stigma is in parentheses. This analysis demonstrates that stigma is a significant mediator between reported CF symptoms and quality of life. *p < .01; **p < .001.

Based on the observed relationships between CF Stigma Scale scores, CF symptom scale scores, and quality of life, we created a model to test the hypothesis that increases in severity of CF symptoms are associated with decreased quality of life and this is mediated by the stigma experience in CF patients (Figure 1). We observed a significant positive relationship between reported symptoms as measured by the CF symptom scale, and experienced stigma, as assessed by the CF Stigma Scale (*p* = 0.003); a significant negative relationship between symptoms and quality of life *(p* < 0.001); and a significant negative relationship between CF stigma and quality of life (*p* < 0.001). Although the relationship between symptoms and quality of life remained significant (*p* < 0.001), accounting for the effects of stigma reduced the magnitude of this relationship. This result indicated partial mediation, whereby the effect of stigma accounted for some, but not all of the variability in quality of life due to experienced symptoms. (*p* = 0.0161). We employed bootstrapping (1000 resamples) owing to the small sample and confirmed a significant effect of mediation (M = -0.26, SE = 0.11, 95% CI -0.51 to -0.08). As Figure 1 illustrates, the unstandardized regression coefficient between CF symptoms and quality of life decreased when controlling for stigma.

## Discussion

Stigma is emerging as an important variable to be considered when working with individuals living with CF. Complex ongoing care, lifelong symptoms, and the inheritable nature of the disease leave adults living with CF vulnerable to the effects of stigma surrounding their disease. This is the first study to investigate stigma in CF and we provide a psychometrically sound tool for evaluating this. Using a mixed-methods design, we demonstrated that quality of life is significantly impacted by patients’ experienced symptoms as a result of their experienced stigma. Comparing our results to Logie and colleagues, [26] we can see that the mean stigma scores in the CF population for the domains of Disclosures and Public attitudes were similar to those for the HIV population (Table 4).

**Table 4:** Comparison of mean CF scores with mean HIV scores

**Domains**	**CF population N = 45 (mean, SD)**	**HIV population N = 173 (mean, SD)**	**p-value**
Overall	16.6 (4.5)	35.3(7.8)	<.001
Sub scales			
Personalized stigma*	1.6 (1.4)	10.6 (3.8)	0.01
Disclosure^#^	2.1 (0.9)	8.6 (2.2)	0.05
Negative self-image^¥^	1.1 (0.5)	7.3 (3.9)	0.03
Public attitudes^$^	2.6 (1.0)	8.4 (2.1)	0.08

Note: *Q’s 7, 8, 9 in CF stigma scale; ^#^Q’s 1 and 10 in CF stigma scale; ^¥^Q’s 2, 3 and 4 in CF stigma scale; ^$^Q’s 5 and 6 in CF stigma scale.

Although our study sample is representative of Canadian adults living with CF, this study was conducted in a single center with a limited number of patients. A multi-center study with patients from different age groups, cultures, ethnicities, occupations, incomes, and educational backgrounds is required to further explore stigma and its impact on those living with CF.

This sample was composed primarily of Caucasian participants (93%) with moderate to high socio-economic status (50% of participants had a household income of ≥ $61,000). Quittner et al. found [27] that CF patients with lower socio-economic status and minority populations experience worse quality of life. Vulnerable population groups might be at a greater risk of experiencing stigma, which in turn may impact their adherence to treatment, health status, and longevity. There is also the question of generalizability of our results to all CF patients. For instance, we did not include patients under the age of 18. It is possible that CF stigma could differ depending on age group (e.g. teenage CF patients may experience more stigma than adults). A validated CF Stigma Scale for use among youth could be a valuable tool for health care professionals who are interested in identifying youth at risk for lower adherence. Despite demonstrating acceptable psychometric properties, our brief CF Stigma Scale needs to be validated in larger populations including different age groups, with different cultures and languages. Furthermore, future studies should include additional CF stigma items which are more relevant to CF symptoms (e.g. stigma related to persistent cough with heavy sputum production).

## Conclusion

Our study found that stigma is an important and measurable construct associated with CF and it significantly impacts the lives of individuals with CF. Although our newly adapted CF Stigma Scale is easy-to-use and has demonstrated acceptable psychometric properties, we believe that it requires further validation in a multi-center study with a more diverse sample of CF patients. Prospective, longitudinal studies are needed to study stigma and its impact on important health outcomes such as treatment adherence, lung function, quality and quantity of life among individuals who are living with CF.

## Competing interests

The authors declare that they have no competing interests.

## Authors’ contributions

This paper is a part of a larger research program entitled, “The Ottawa Cystic Fibrosis Treatment Knowledge and Adherence Program”. Drs. SP, LB, SA, GT and BC are responsible for study concept, study design, creation of the tools used in the study such as the Cystic Fibrosis Stigma Scale which is validated here. Ms. EG, Mr. MA, Dr. CH and Dr. RE were responsible for data collection, data cleaning, data entry, data analysis for this study. All authors made substantial contribution to the interpretation of the data, revised the article critically for important intellectual content, and gave their final approval of the version to be published.

## Pre-publication history

The pre-publication history for this paper can be accessed here:

http://www.biomedcentral.com/1471-2466/14/76/prepub

## Notation of prior abstract publication/presentation

Some data from this study was presented at the North American CF Conference (NACFC) – 2011 in Anaheim, California and the American Thoracic Society (ATS) Conference - 2013 in Philadelphia, Pennsylvania in abstract form.
