# Correction to: Assessment of stigma in patients with cystic fibrosis

**DOI:** 10.1186/s12890-020-1176-0

**Published:** 2020-05-20

**Authors:** Smita Pakhale, Michael Armstrong, Crystal Holly, Rojiemiahd Edjoc, Ena Gaudet, Shawn Aaron, Giorgio Tasca, William Cameron, Louise Balfour

**Affiliations:** 1 Ottawa Hospital Research Institute, 501 Smyth Road, Ottawa, Ontario K1H 8 M5 Canada; 2 The Ottawa Hospital, 501 Smyth Road, Ottawa, Ontario K1H 8 M5 Canada; 3 The University of Ottawa, 451 Smyth Road, Ottawa, Ontario K1H 8 M5 Canada; 4 Division of Respiratory Medicine, The Ottawa Hospital, 501 Smyth Road, Ottawa, Ontario K1H 8 L6 Canada

**Correction to: BMC Pulm Med (2014) 14:76**

**https://doi.org/10.1186/1471-2466-14-76**

Following publication of the original article [1], the authors reported an error in Table 2 of the article.

Namely, rows ‘2.’ and ‘9.’ are missing from the table.

Please now see (the correct version of) Table 2 provided in this correction.

**Table 2:** The baseline CF Stigma Scale scores

*N* = 45	Strongly Disagree = 1 N(%)	Disagree = 2 N(%)	Agree = 3 N(%)	Strongly Agree = 4 N(%)
1. I am very careful who I tell that I have CF.	6 (13.3)	17 (37.8)	10 (22.2)	11 (24.4)
2. I feel that I am not as good a person as others because I have CF.	19 (42.2)	19 (42.2)	5 (11.1)	1 (2.2)
3. Having CF makes me feel unclean.	30 (66.7)	7 (15.6)	6 (13.3)	1 (2.2)
4. Having CF makes me feel that I’m a bad person.	37 (82.2)	6 (13.3)	1 (2.2)	–
5. Most people think that a person with CF is disgusting.	25 (55.6)	13 (28.9)	6 (13.3)	–
6. Most people with CF are rejected when others find out.	23 (51.1)	19 (42.2)	2 (4.4)	–
7. I have been hurt by how people reacted to learning I have CF.	22 (48.9)	11 (24.4)	2 (4.4)	1 (2.2)
8. I have stopped socializing with some people because of their reactions to my having CF.	23 (51.1)	13 (28.9)	7 (15.6)	–
9. I have lost friends by telling them I have CF.	27 (60.0)	15 (33.3)	2 (4.4)	–
10. I worry that people who know I have CF will tell others.	23 (51.1)	15 (33.3)	4 (8.9)	2 (4.4)

Note: Higher agreement reflects higher stigma experienced by patient

## References

[1] Pakhale S, Armstrong M, Holly C (2014). Assessment of stigma in patients with cystic fibrosis. BMC Pulm Med.

